# Predictors of clinical outcomes in Chinese patients with carbapenem-resistant Gram-negative bacterial infections receiving colistimethate sodium: a retrospective cohort study

**DOI:** 10.3389/fcimb.2026.1824625

**Published:** 2026-05-20

**Authors:** Jianpan Zhang, Huaidong Peng, Jiajia Liao, Ruolun Wang, Xiaoli Wu, Lijin Chen

**Affiliations:** Department of Pharmacy, The Second Affiliated Hospital, Guangzhou Medical University, Guangzhou, China

**Keywords:** carbapenem-resistant Gram-negative bacteria, clinical outcomes, colistimethate sodium, nebulization, predictors

## Abstract

**Objective:**

To evaluate the clinical outcomes of colistimethate sodium (CMS) in Chinese patients with carbapenem-resistant Gram-negative bacterial (CR-GNB) infections and to identify predictors associated with clinical outcomes.

**Methods:**

A retrospective cohort study was performed in patients with CR-GNB infections who received CMS treatment between April 2022 and December 2025. The primary outcome was clinical efficacy; secondary outcomes included bacterial eradication and all-cause mortality. Predictors were identified using multivariable logistic and Cox regression.

**Results:**

A total of 169 patients were included. The clinical efficacy was 40.8%, microbiological eradication was 50.3%, and all-cause mortality was 36.7%. Multivariate analysis showed that colistin MIC ≤ 0.5 mg/L (adjusted odds ratio [aOR] 3.495; 95% confidence interval [CI] 1.279–9.554; *p* = 0.015) and duration of CMS therapy (aOR 1.143; 95% CI 1.062–1.229; *p* < 0.001) were independently associated with favorable clinical efficacy, whereas vasoactive agent use (aOR 0.183; 95% CI 0.062–0.538; *p* = 0.002) and CMS monotherapy (aOR 0.076; 95% CI 0.008–0.706; *p* = 0.024) were inversely correlated with clinical success. Patients treated for 14–21 days with a cumulative CMS dose of 40–50 mg CBA/kg tended to have more favorable clinical outcomes. Combination with nebulization (adjusted hazard ratio [aHR] 0.416; 95% CI 0.246–0.703; *p* = 0.001) and concomitant β−lactam/β−lactamase inhibitor combinations (aHR 0.556; 95% CI 0.316–0.978; *p* = 0.042) were associated with lower all-cause mortality.

**Conclusion:**

CMS demonstrated potential therapeutic value in the treatment of CR-GNB infections. Longer treatment durations (particularly 14–21 days) and combination therapy were independent predictors of higher clinical efficacy. Adjunctive nebulized CMS therapy correlated with reduced all-cause mortality, particularly in patients with respiratory tract infections.

## 1 Introduction

The widespread dissemination of carbapenem-resistant Gram-negative bacteria (CR-GNB) has emerged as a critical global public health challenge (Macesic et al., 2025; Murray et al., 2022; World Health Organization, 2024). CR-GNB typically exhibit resistance to multiple antimicrobial agents, posing major challenges to clinical treatment. This not only leads to substantial consumption of healthcare resources but also significantly reduces the effectiveness of infection management (GBD 2021 Antimicrobial Resistance Collaborators, 2024). According to the 2024 data from the China Antimicrobial Surveillance Network (CHINET), the detection rates of carbapenem-resistant *Acinetobacter baumannii* (CRAB), carbapenem-resistant *Klebsiella pneumoniae* (CRKP), and carbapenem-resistant *Pseudomonas aeruginosa* (CRPA) were 64.7%, 21.2%, and 17.3%, respectively (CHINET China Antimicrobial Surveillance Network, 2024). Several novel antimicrobial agents have recently been approved in China, but their clinical use remains limited by restricted hospital availability and high costs. Polymyxins, therefore, remain a critical treatment option for patients with severe CR-GNB infections (Zeng et al., 2023; Ding and Hu, 2023; Luo et al., 2024).

Polymyxins, which exert their antibacterial effect by disrupting the outer membrane integrity of Gram-negative bacteria, were introduced into clinical use in the 1950s (Diani et al., 2024). However, their use declined due to the advent of safer alternative antimicrobial agents and concerns regarding nephrotoxicity and neurotoxicity (Mousavi et al., 2025; Nang et al., 2021). In recent years, faced with escalating antimicrobial resistance and limited therapeutic options, polymyxins have regained their role as essential agents against CR-GNB infections (Huang et al., 2024). In China, the available polymyxin formulations include polymyxin B, colistimethate sodium (CMS), and polymyxin E sulfate. As the latest agent in this class to enter the Chinese clinical setting, CMS has not yet been extensively evaluated in the local population, leading to a relative lack of local clinical experience compared with the well-established international evidence (Son et al., 2024; Ahumada Topete et al., 2023). In real-world clinical practice, CMS dosing strategies often vary considerably, influenced by clinician experience and concerns regarding potential toxicity. As a prodrug, CMS is slowly converted to active colistin *in vivo* with limited lung penetration, both of which raise concerns about suboptimal drug exposure and treatment failure. Given that the role of adjunctive strategies such as nebulization remains controversial, the optimal combination of CMS dosing regimens and treatment duration in real-world practice remains unclear for patients with CR-GNB infections. Further investigation into the clinical efficacy, safety, and optimal dosing strategies of CMS in Chinese populations is urgently warranted.

The present study aimed to evaluate the clinical efficacy of CMS in Chinese patients with CR-GNB infections. The primary objective was to identify independent predictors of clinical efficacy, with a particular focus on the impact of treatment duration and real-world dosing practices. Secondary objectives included evaluating the impact of adjunctive nebulization on all-cause mortality and characterizing the safety profile of CMS in this population. The study aims to provide clinical insights into optimizing CMS therapeutic regimens and improving outcomes for patients with CR-GNB infections.

## 2 Materials and methods

### 2.1 Study design

This retrospective cohort study was conducted at the Second Affiliated Hospital of Guangzhou Medical University between April 2022 and December 2025. The study protocol was approved by the Institutional Ethics Committee (Registration number: 2024-hg-ks-22). Inclusion criteria were: (1) age ≥ 18 years; (2) microbiologically confirmed CR-GNB infection; and (3) intravenous CMS (Chia Tai Tianqing Pharmaceutical Group Co., Ltd., China) treatment for ≥ 72 hours. Exclusion criteria were: (1) pregnancy or lactation; (2) known hypersensitivity to polymyxins; or (3) incomplete medical records.

### 2.2 Data collection

Clinical data were extracted from electronic medical records and categorized as follows: (1) baseline characteristics, including age, sex, body weight, Acute Physiology and Chronic Health Evaluation II (APACHE II) score, Charlson Comorbidity Index (CCI), underlying comorbidities, and respiratory support requirements; (2) microbiological data, comprising infection sites, identified pathogens, and minimum inhibitory concentrations (MICs) of colistin; and (3) treatment regimens, detailing timing of therapy initiation, loading and maintenance doses, administration routes, treatment duration, and concomitant antimicrobial agents.

### 2.3 Outcomes and definitions

The primary outcome was clinical efficacy. Secondary outcomes included bacterial eradication, all-cause mortality, and safety. Clinical efficacy was defined as meeting all the following criteria: hemodynamic stability (systolic blood pressure > 90 mmHg without vasoactive agents), sustained defervescence (temperature < 37.5 °C for ≥ 72 hours), resolution of infection-related symptoms, and significant recovery of inflammatory markers. Physician-documented improvement was also required, with assessment strictly based on the above objective clinical and laboratory indicators (Weiss et al., 2019). Treatment failure was defined as a lack of clinical improvement, or discontinuation of therapy due to either refractory hypotension or drug intolerance (Qu et al., 2022). Microbiological eradication was defined as the negative conversion of cultures from the original infection site following CMS therapy completion. In cases of polymicrobial infections, the failure to clear any baseline pathogen was classified as non-eradication (Pirnay et al., 2024). All-cause mortality was defined as death from any cause during hospitalization, including cases of withdrawal of life-sustaining therapy. All outcome assessments were independently conducted by two researchers to reduce assessment bias.

Safety assessment focused primarily on CMS-associated nephrotoxicity. For this specific analysis, patients receiving renal replacement therapy (RRT) prior to CMS initiation or those with missing baseline serum creatinine (SCr) data were excluded. AKI was defined and staged according to the Kidney Disease: Improving Global Outcomes (KDIGO) criteria (KDIGO Acute Kidney Injury Work Group, 2012). Due to the lack of hourly urine output records, AKI diagnosis relied exclusively on SCr changes. Baseline SCr was defined as the most recent measurement prior to the first CMS dose. AKI was identified as: (1) an increase in SCr of ≥ 0.3 mg/dL (≥ 26.5 μmol/L) within 48 hours; or (2) an increase in SCr to ≥ 1.5 times baseline within 7 days. Patients requiring new-onset RRT during treatment were classified as stage 3 AKI.

### 2.4 Microbiology

Pathogen isolation and identification were performed per standard clinical laboratory protocols. Bacterial identification and routine antimicrobial susceptibility testing were carried out using the VITEK 2 Compact automated system (bioMérieux, Marcy-l’Étoile, France). Specifically, colistin susceptibility was determined using the broth microdilution method. The results were interpreted according to the breakpoints established by the United States Committee on Antimicrobial Susceptibility Testing (USCAST, version 7.0, 2023), defining susceptibility as an MIC ≤ 2 mg/L (Pogue et al., 2020).

### 2.5 Statistical analysis

Data were analyzed using SPSS version 27.0 (IBM Corp., Armonk, NY, USA). Normally distributed continuous variables were presented as mean ± standard deviation (SD), and comparisons between groups were performed using the Student’s t-test. Non-normally distributed continuous variables were described as median (interquartile range [IQR], Q1–Q3) and analyzed with the Mann-Whitney U test. Categorical variables were expressed as frequencies and percentages (n [%]), and intergroup comparisons were performed using the chi-square test or Fisher’s exact test, as appropriate.

Patients were stratified by treatment duration to compare clinical efficacy rates among different subgroups. Multivariable binary logistic regression was used to identify independent predictive factors for clinical efficacy and bacterial eradication. The Kaplan–Meier method and log−rank test were adopted for survival analysis of all−cause mortality, and Cox proportional hazards regression was performed to evaluate mortality−related risk factors. To minimize confounding bias, multivariable models included variables with significant differences in univariate analysis (*p* < 0.05) and key covariates with clear clinical relevance. The number of included variables was restricted according to event counts to prevent model overfitting. Collinearity diagnostics, model fit and discriminative performance were assessed for all regression analyses. Variance inflation factor (VIF < 5) was applied to detect collinearity in logistic models; the Hosmer–Lemeshow test (*p* > 0.05) and the area under the curve (AUC) were adopted to evaluate model calibration and discrimination. For Cox regression, Spearman correlation coefficients (|r| < 0.7) were used to detect collinearity among independent variables, and the Schoenfeld residual test was performed to verify the proportional hazards assumption. Stratified Cox regression was utilized for variables that violated this assumption. All statistical tests were two−tailed, and a *p*−value < 0.05 was defined as statistically significant.

## 3 Results

### 3.1 Baseline demographic and clinical data

**Figure 1:**
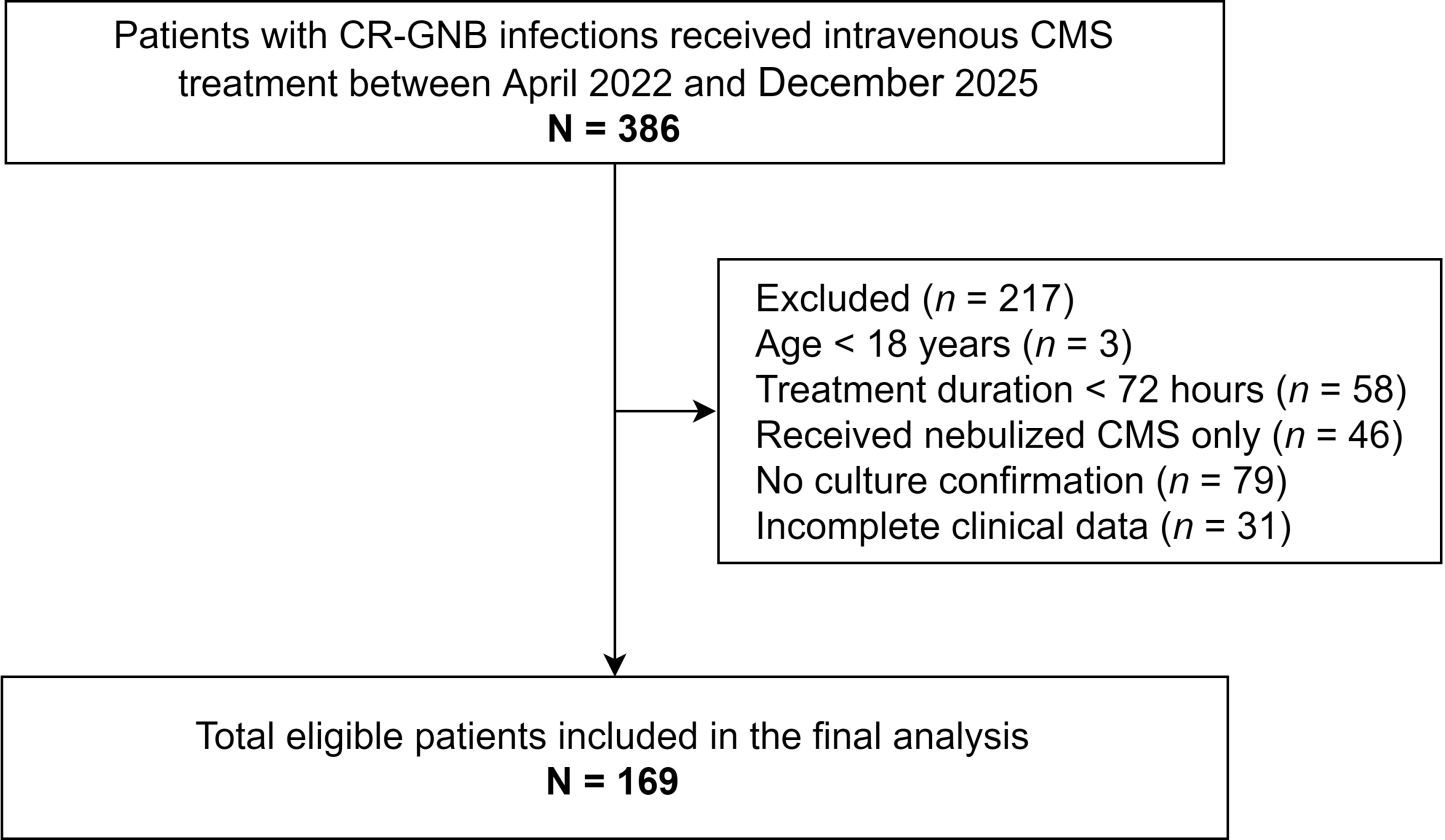
Flowchart of patient selection.

A total of 169 patients were enrolled in this study (**Figure 1**). The cohort consisted predominantly of males (121, 71.6%), with a median age of 72.0 years (63.0–80.5) and a median body weight of 61.0 kg (52.0–68.0). Most patients (150, 88.8%) were admitted to the intensive care unit (ICU). The median APACHE II score was 19 (15–25), and the median age-adjusted CCI was 7.0 (5.0–8.5). Mechanical ventilation was required in 139 (82.2%) patients, and vasoactive agents were administered to 121 (71.6%). Comorbidities included chronic respiratory diseases (84, 49.7%), diabetes mellitus (62, 36.7%) and hypertension (99, 58.6%). Additionally, 27 (16.0%) patients had malignancy, and 39 (23.1%) presented with serum albumin < 25 g/L (**Table 1**).

**Table 1:** Demographic and clinical characteristics of the included patients.

Variables	Total (n = 169)
Age (years)	72.0 (63.0-80.5)
Sex (male)	121 (71.6%)
Weight (kg)	61.0 (52.0-68.0)
ICU admission	150 (88.8%)
APACHE II	19 (15-25)
Charlson index	7.0 (5.0-8.5)
Vasoactive agents	121 (71.6%)
Mechanical ventilation	139 (82.2%)
Serum Albumin < 25 g/L	39 (23.1%)
Comorbidities	
Chronic respiratory disease	84 (49.7%)
Diabetes mellitus	62 (36.7%)
Hypertension	99 (58.6%)
Malignancy	27 (16.0%)
Infection site	
Respiratory tract	163 (96.4%)
Bloodstream	29 (17.2%)
Abdominal	7 (4.1%)
Urinary tract	14 (8.3%)
Infection site (≥ 2)	34 (20.1%)
Microbiology	
CRAB	85 (50.3%)
CRKP	81 (47.9%)
CRPA	37 (21.9%)
Number of CR-GNB (≥ 2)	40 (23.7%)
Colistin MIC ≤ 0.5 mg/L	119 (70.4%)
Treatment Regimens	
Time to CMS initiation ≤ 24 h	105 (62.1%)
Loading dose administered	47 (27.8%)
Maintenance dose (mg CBA/kg/day)	2.8 (2.3-3.8)
Combined nebulization	99 (58.6%)
Cumulative dose (mg CBA/kg)	27.3 (17.4-40.8)
Duration of CMS therapy (days)	9.0 (6.0-13.5)
Monotherapy	22 (13.0%)

Vasoactive agents include epinephrine, norepinephrine, isoproterenol, and dopamine.

ICU, Intensive Care Unit; APACHE II, Acute Physiology and Chronic Health Evaluation II; CRAB, carbapenem-resistant *Acinetobacter baumannii*; CRKP, carbapenem-resistant *Klebsiella pneumoniae*; CRPA, carbapenem-resistant *Pseudomonas aeruginosa*; CR-GNB, carbapenem-resistant Gram-negative bacteria; MIC, minimum inhibitory concentration; CBA, Colistin base activity; CMS, Colistimethate Sodium.

### 3.2 Microbiological characteristics

Respiratory tract infections were the most prevalent (163, 96.4%), followed by bloodstream (29, 17.2%), urinary tract (14, 8.3%), and intra-abdominal infections (7, 4.1%). Multiple-site infections were observed in 34 (20.1%) patients. The most prevalent pathogens were CRAB (85, 50.3%), CRKP (81, 47.9%), and CRPA (37, 21.9%). Co-infection with two or more CR-GNB strains was identified in 40 (23.7%) patients.

### 3.3 CMS treatment and combination therapy regimens

Isolates from 119 (70.4%) patients exhibited colistin MIC of ≤ 0.5 mg/L. CMS therapy was initiated within 24 hours of CR-GNB detection in 105 (62.1%) patients, and loading doses were administered to 47 (27.8%). The median maintenance dose was 2.8 mg CBA/kg/day (2.3–3.8), with a median cumulative dose of 27.3 mg CBA/kg (17.4–40.8). Adjunctive nebulization was utilized in 99 (58.6%) patients. Most patients (147, 87.0%) received combination therapy, while monotherapy was prescribed in only 22 (13.0%). The median duration of CMS treatment was 9.0 (6.0–13.5) days.

### 3.4 Clinical outcomes

As detailed in **Table 2**, the median length of ICU stay was 28.0 (14.5–42.0) days, and the median total duration of hospitalization was 34.0 (22.5–53.5) days. Clinical efficacy was achieved in 69 (40.8%) patients, and microbiological eradication was achieved in 85 (50.3%). The 28-day and all-cause mortality rates were 32.0% (54/169) and 36.7% (62/169), respectively. Among the 120 patients included in the safety analysis, AKI occurred in 50 (41.7%). The distribution of AKI severity was predominantly mild, with 32 (64.0%), 6 (12.0%), and 12 (24.0%) patients classified as Stage 1, Stage 2, and Stage 3, respectively.

**Table 2:** Clinical outcomes and adverse events of the included patients.

Variables	Total (n = 169)
Outcomes	
Length of ICU stay (days)	28.0 (14.5-42.0)
Length of hospital stay (days)	34.0 (22.5-53.5)
Clinical efficacy	69 (40.8%)
Bacterial eradication after treatment	85 (50.3%)
28-day mortality	54 (32.0%)
All-cause mortality	62 (36.7%)
Acute kidney injury	50 (41.7%)
KDIGO Stage 1	32 (64.0%)
KDIGO Stage 2	6 (12.0%)
KDIGO Stage 3	12 (24.0%)

ICU, Intensive Care Unit; KDIGO, Kidney Disease: Improving Global Outcomes.

### 3.5 Predictors of clinical outcomes and mortality

#### 3.5.1 Predictors of clinical efficacy

**Table 3** details the univariate and multivariate logistic regression analyses for clinical efficacy. Univariate analysis identified several factors significantly associated with treatment outcomes, including APACHE II score, use of vasoactive agents, serum albumin < 25 g/L, colistin MIC ≤ 0.5 mg/L, duration of CMS therapy, CMS monotherapy, and combination with β-lactam/β-lactamase inhibitors (BL/BLIs).

**Table 3:** Univariate and multivariate logistic regression analyses for clinical efficacy in included patients.

Variables	Univariate analysis			Multivariable analysis	
	Effective (n = 69)	Ineffective (n = 100)	*p*	Adjusted OR (95% CI)	*p*
Age (years)	70.0 (62.3-79.8)	73.0 (64.0-81.0)	0.114		
Sex (male)	48 (69.6%)	73 (73.0%)	0.626		
Weight (kg)	62.64 ± 12.28	59.90 ± 12.08	0.153		
ICU admission	59 (85.5%)	91 (91.0%)	0.267		
APACHE II	17.5 (13.0-23.8)	20.0 (16.0-25.0)	**0.020**	0.945 (0.888–1.005)	0.072
Charlson index	6.0 (5.0-8.0)	7.0 (5.3-8.8)	0.082		
Vasoactive agents^a^	40 (58.0%)	81 (81.0%)	**0.001**	0.183 (0.062–0.538)	**0.002**
Mechanical ventilation	52 (75.4%)	87 (87.0%)	0.052		
Serum Albumin < 25 g/L	8 (11.6%)	31 (31.0%)	**0.003**	0.560 (0.195–1.611)	0.282
Comorbidities					
Chronic respiratory disease	34 (49.3%)	50 (50.0%)	0.926		
Diabetes mellitus	24 (34.8%)	38 (38.0%)	0.670		
Hypertension	36 (52.2%)	63 (63.0%)	0.160		
Cardiovascular disease	32 (46.4%)	54 (54.0%)	0.330		
Malignancy	9 (13.0%)	18 (18.0%)	0.387		
Infection site					
Bloodstream	11 (15.9%)	18 (18.0%)	0.727		
Abdominal	4 (5.8%)	3 (3.0%)	0.303		
Urinary tract	5 (7.2%)	9 (9.0%)	0.684		
Infection site (≥ 2)	13 (18.8%)	21 (21.0%)	0.731		
Microbiology					
CRAB	36 (52.2%)	49 (49.0%)	0.685		
CRKP	29 (42.0%)	52 (52.0%)	0.202		
CRPA	16 (23.2%)	21 (21.0%)	0.735		
Other CR-GNB	7 (10.1%)	5 (5.0%)	0.201		
Number of CR-GNB (≥ 2)	18 (26.1%)	22 (22.0%)	0.539		
Colistin MIC ≤ 0.5 mg/L	55 (79.7%)	64 (64.0%)	**0.028**	3.495 (1.279–9.554)	**0.015**
Treatment Regimens					
Time to CMS initiation ≤ 24 h	48 (69.6%)	57 (57.0%)	0.098		
Loading dose administered	14 (20.3%)	33 (33.0%)	0.070		
Maintenance dose ≥ 2.5 mg CBA/kg/day	44 (63.8%)	61 (61.0%)	0.715		
Maintenance dose (mg CBA/kg/day)	2.8 (2.3-3.8)	2.9 (2.3-3.8)	0.663		
Combined nebulization	45 (65.2%)	54 (54.0%)	0.146		
Duration of CMS therapy (days)	12.5 (9.0-15.8)	7.0 (5.0-11.8)	**< 0.001**	1.143 (1.062–1.229)	**< 0.001**
Monotherapy	4 (5.8%)	18 (18.0%)	**0.016**	0.076 (0.008–0.706)	**0.024**
Combined with Carbapenems	33 (47.8%)	50 (50.0%)	0.781		
Combined with BL/BLI	34 (49.3%)	32 (32.0%)	**0.024**		
Combined with Tigecycline	10 (14.5%)	13 (13.0%)	0.781		

a. Vasoactive agents include epinephrine, norepinephrine, isoproterenol, and dopamine. Bold values represent statistically significant associations (*p* < 0.05).

ICU, Intensive Care Unit; APACHE II, Acute Physiology and Chronic Health Evaluation II; CRAB, carbapenem-resistant *Acinetobacter baumannii*; CRKP, carbapenem-resistant *Klebsiella pneumoniae*; CRPA, carbapenem-resistant *Pseudomonas aeruginosa*; CR-GNB, carbapenem-resistant Gram-negative bacteria; MIC, minimum inhibitory concentration; CMS, Colistimethate Sodium; CBA, colistin base activity; OR, odds ratio; CI, confidence interval. BL/BLI, β-lactam/β-lactamase inhibitor.

**Figure 2:**
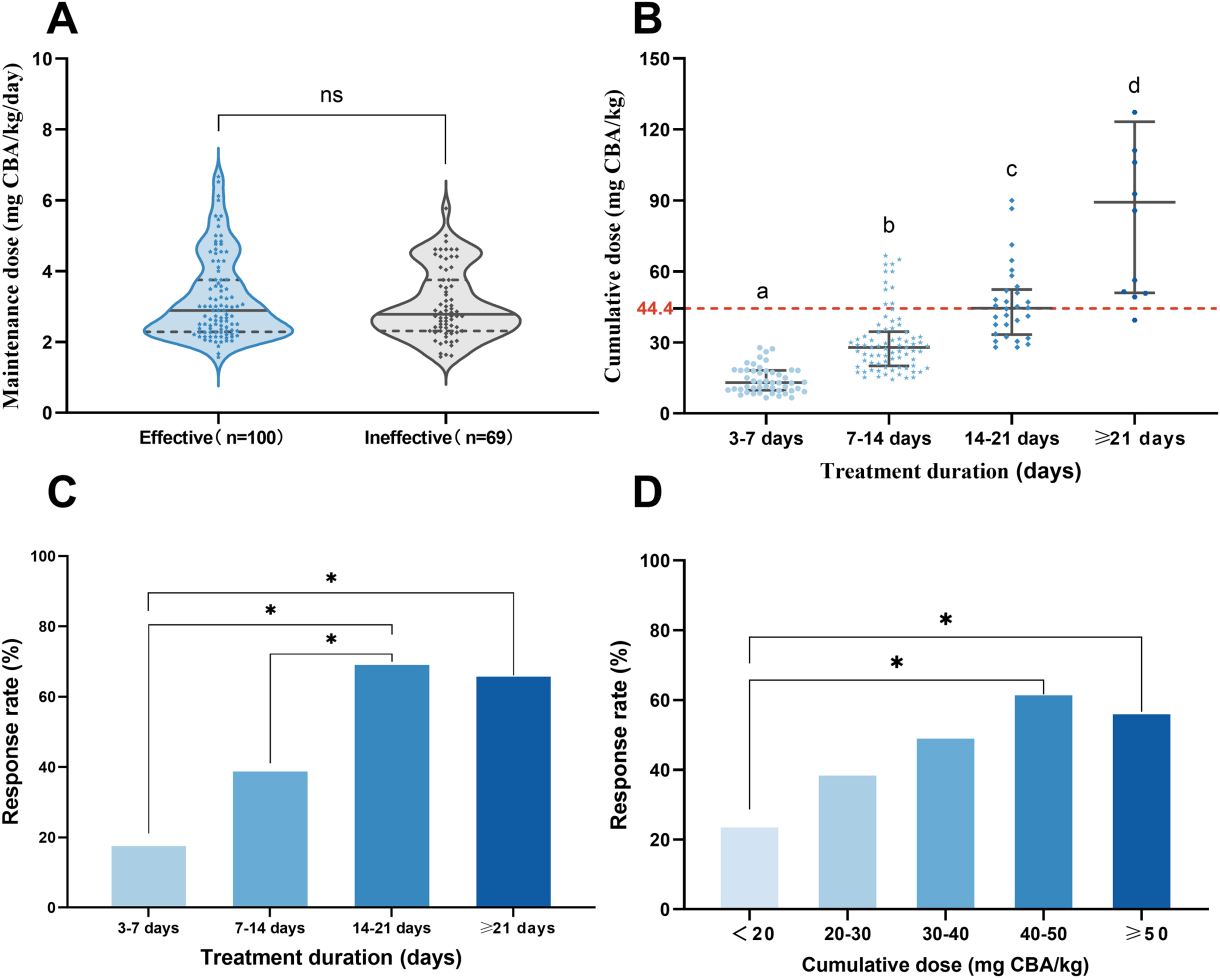
Impact of dosage and treatment duration on clinical response to CMS treatment. **(A)** Maintenance doses in the effective (n = 100) and ineffective (n = 69) groups (ns, not significant). **(B)** Cumulative doses across treatment durations. The red dashed line indicates the 44.4 mg/kg cutoff. Different letters (a–d) indicate significant differences. **(C, D)** The clinical response rates stratified by treatment duration **(C)** and cumulative dose **(D)**. *p < 0.0083 (Bonferroni correction). CMS, colistimethate sodium.

In the multivariate analysis, lower colistin MIC (≤ 0.5 mg/L) (adjusted odds ratio [aOR] 3.495; 95% confidence interval [CI] 1.279–9.554; *p* = 0.015) and duration of CMS therapy (aOR 1.143; 95% CI 1.062–1.229; *p* < 0.001) were independently correlated with clinical efficacy. Conversely, the use of vasoactive agents (aOR 0.183; 95% CI 0.062–0.538; *p* = 0.002) and CMS monotherapy (aOR 0.076; 95% CI 0.008–0.706; *p* = 0.024) showed independent links to treatment failure. Subgroup analysis further revealed that more favorable clinical outcomes were observed in patients with a treatment duration of 14–21 days and a cumulative CMS dose of 40–50 mg CBA/kg (**Figure 2**).

#### 3.5.2 Predictors of microbiological eradication

As shown in **Table 4**, univariate analysis detected several factors associated with bacterial eradication, including male sex, bloodstream infection, multiple infection sites, pathogenic species (CRAB and CRKP), colistin MIC ≤ 0.5 mg/L, duration of CMS therapy and combination regimens (with BL/BLIs or tigecycline).

**Table 4:** Univariate and multivariate logistic regression analyses for bacterial eradication.

Variables	Univariate analysis			Multivariable analysis	
	Non-eradication isolates (n=84)	Eradication isolates (n=85)	*p*	OR (95% CI)	*p*
Age (years)	71.0 (62.5-80.5)	73.0 (65.0-81.0)	0.361		
Sex (male)	68 (81.0%)	53 (62.4%)	**0.007**	0.310 (0.140–0.684)	0.004
Weight (kg)	62.0 (51.0-70.0)	60.0 (50.0-67.0)	0.202		
ICU admission	71 (84.5%)	79 (92.9%)	0.083		
APACHE II	19.39 ± 6.08	20.71 ± 7.50	0.252		
Charlson index	7.0 (5.0-8.0)	7.0 (5.0-9.0)	0.360		
Vasoactive agents^a^	59 (70.2%)	62 (72.9%)	0.697		
Mechanical ventilation	66 (78.6%)	73 (85.9%)	0.214		
Serum Albumin < 25 g/L	21 (25.0%)	18 (21.2%)	0.555		
Comorbidities					
Chronic respiratory disease	40 (47.6%)	44 (51.8%)	0.590		
Diabetes mellitus	33 (39.3%)	29 (34.1%)	0.486		
Hypertension	49 (58.3%)	50 (58.8%)	0.948		
Cardiovascular disease	43 (51.2%)	43 (50.6%)	0.938		
Malignancy	14 (16.7%)	13 (15.3%)	0.808		
Infection site					
Bloodstream	20 (23.8%)	9 (10.6%)	**0.023**	0.259 (0.096–0.696)	**0.007**
Abdominal	2 (2.4%)	5 (5.9%)	0.227		
Urinary tract	9 (10.7%)	5 (5.9%)	0.255		
Infection site (≥ 2)	22 (26.2%)	12 (14.1%)	**0.038**		
Microbiology					
CRAB	31 (36.9%)	54 (63.5%)	**< 0.001**	2.780 (1.354–5.709)	**0.005**
CRKP	52 (61.9%)	29 (34.1%)	**< 0.001**		
CRPA	23 (27.4%)	14 (16.5%)	0.086		
Other CR-GNB	5 (6.0%)	7 (8.2%)	0.563		
Colistin MIC ≤ 0.5 mg/L	51 (60.7%)	68 (80.0%)	**0.006**	2.156 (0.950–4.891)	0.066
Treatment Regimens					
Time to CMS initiation ≤ 24 h	55 (65.5%)	50 (58.8%)	0.373		
Loading dose administered	20 (23.8%)	27 (31.8%)	0.248		
Maintenance dose ≥ 2.5 mg CBA/kg/day	51 (60.7%)	54 (63.5%)	0.706		
Maintenance dose (mg CBA/kg/day)	2.9 (2.3-3.9)	2.8 (2.3-3.6)	0.910		
Combined Nebulization	45 (53.6%)	54 (63.5%)	0.189		
Duration of CMS therapy (days)	7.0 (4.5-12.0)	11.0 (7.0-15.0)	**< 0.001**	1.036 (0.980–1.095)	0.209
Previous use of carbapenem	38 (45.2%)	43 (50.6%)	0.486		
Previous use of BL/BLI	65 (77.4%)	64 (75.3%)	0.750		
Previous use of quinolone	16 (19.0%)	16 (18.8%)	0.970		
Previous use of tigecycline	10 (11.9%)	11 (12.9%)	0.838		
Monotherapy	10 (11.9%)	12 (14.1%)	0.669		
Combined with Carbapenems	47 (56.0%)	36 (42.4%)	0.077		
Combined with BL/BLI	26 (31.0%)	40 (47.1%)	**0.032**	2.367 (1.135–4.938)	**0.022**
Combined with Tigecycline	7 (8.3%)	16 (18.8%)	**0.047**	2.991 (1.040–8.601)	**0.042**

a. Vasoactive agents include epinephrine, norepinephrine, isoproterenol, and dopamine. Bold values represent statistically significant associations (*p* < 0.05)

ICU, Intensive Care Unit; APACHE II, Acute Physiology and Chronic Health Evaluation II; CRAB, carbapenem-resistant *Acinetobacter baumannii*; CRKP, carbapenem-resistant *Klebsiella pneumoniae*; CRPA, carbapenem-resistant *Pseudomonas aeruginosa*; CR-GNB, carbapenem-resistant Gram-negative bacteria; MIC, minimum inhibitory concentration; CMS, Colistimethate Sodium; CBA, colistin base activity; OR, odds ratio; CI, confidence interval; BL/BLI, β-lactam/β-lactamase inhibitor.

Multivariate analysis showed that infection with CRAB (aOR 2.780; 95% CI 1.354–5.709; *p* = 0.005), combination with tigecycline (aOR 2.991; 95% CI 1.040–8.601; *p* = 0.042) and combination with BL/BLIs (aOR 2.367; 95% CI 1.135–4.938; *p* = 0.022) were independently associated with higher eradication. In contrast, male sex (aOR 0.310; 95% CI 0.140–0.684; *p* = 0.004) and bloodstream infection (aOR 0.259; 95% CI 0.096–0.696; *p* = 0.007) were inversely associated with microbiological eradication.

#### 3.5.3 Predictors of all-cause mortality

Univariate Cox proportional hazards analyses for all−cause mortality are detailed in **Table 5**. In the univariate analysis, significant factors associated with mortality included serum albumin < 25 g/L, colistin MIC ≤ 0.5 mg/L, Vasoactive agents, and adjunctive nebulization. Combination with BL/BLIs showed borderline significance (*p* = 0.053) and was therefore retained in the multivariate model as a clinically relevant variable.

**Table 5:** Univariate cox proportional hazards regression analysis for all-cause mortality.

Variables	Survivors (n= 107)	Non-survivors (n= 62)	Univariate HR (95% CI)	*p*
Age (per year)	70.0 (60.3-80.0)	74.0 (68.0-82.5)	1.016 (0.996-1.035)	0.115
Sex (male)	78 (72.9%)	43 (69.4%)	0.872 (0.507-1.498)	0.619
Weight (kg)	62.0 (53.0-68.0)	60.0 (50.0-70.0)	1.002 (0.982-1.023)	0.822
ICU admission	93 (86.9%)	57 (91.9%)	0.807 (0.317-2.051)	0.652
APACHE II	18.0 (15.0-25.0)	21.0 (17.0-24.8)	1.012 (0.975-1.049)	0.541
Charlson index	7.0 (5.0-8.0)	7.0 (6.0-8.0)	1.012 (0.931-1.099)	0.782
Vasoactive agents^a^	65 (60.7%)	56 (90.3%)	3.028 (1.295-7.077)	**0.011**
Mechanical ventilation	83 (77.6%)	56 (90.3%)	2.013 (0.864-4.687)	0.105
Serum Albumin < 25 g/L	14 (13.1%)	25 (40.3%)	2.224 (1.337-3.700)	**0.002**
Comorbidities				
Chronic respiratory disease	52 (48.6%)	32 (51.6%)	1.039 (0.630-1.713)	0.882
Diabetes mellitus	38 (35.5%)	24 (38.7%)	1.012 (0.602-1.699)	0.965
Hypertension	61 (57.0%)	38 (61.3%)	1.281 (0.768-2.138)	0.343
Cardiovascular disease	51 (47.7%)	35 (56.5%)	1.035 (0.624-1.715)	0.894
Malignancy	18 (16.8%)	9 (14.5%)	1.273 (0.624-2.597)	0.507
Infection site				
Bloodstream	15 (14.0%)	14 (22.6%)	1.185 (0.651-2.154)	0.579
Abdominal	5 (4.7%)	2 (3.2%)	1.007 (0.245-4.136)	0.993
Urinary tract	8 (7.5%)	6 (9.7%)	1.243 (0.530-2.915)	0.617
Infection site (≥ 2)	17 (15.9%)	17 (27.4%)	1.292 (0.738-2.261)	0.370
Microbiology				
CRAB	53 (49.5%)	32 (51.6%)	0.907 (0.551-1.495)	0.703
CRKP	50 (46.7%)	31 (50.0%)	1.039 (0.631-1.710)	0.881
CRPA	24 (22.4%)	13 (21.0%)	0.774 (0.417-1.436)	0.416
Other CR-GNB	9 (8.4%)	3 (4.8%)	0.394 (0.123-1.259)	0.116
Number of CR-GNB (≥ 2)	26 (24.3%)	14 (22.6%)	0.779 (0.429-1.414)	0.411
Colistin MIC ≤ 0.5mg/L	79 (73.8%)	40 (64.5%)	0.583 (0.344-0.986)	**0.044**
Treatment Regimens				
Time to CMS initiation ≤ 24 h	69 (64.5%)	36 (58.1%)	1.298 (0.782-2.154)	0.314
Loading dose administered	27 (25.2%)	20 (32.3%)	1.645 (0.955-2.832)	0.073
Maintenance dose ≥ 2.5 mg CBA/kg/day	67 (62.6%)	38 (61.3%)	0.956 (0.573-1.595)	0.863
Maintenance dose (mg CBA/kg/day)	2.8 (2.3-3.8)	2.7 (2.3-3.8)	1.095 (0.875-1.371)	0.427
Combined Nebulization	69 (64.5%)	30 (48.4%)	0.452 (0.272-0.750)	**0.002**
Monotherapy	14 (13.1%)	8 (12.9%)	1.308 (0.615-2.781)	0.485
Combined with Carbapenems	48 (44.9%)	35 (56.5%)	1.053 (0.634-1.750)	0.842
Combined with BL/BLI	48 (44.9%)	18 (29.0%)	0.580 (0.335-1.007)	0.053
Combined with Tigecycline	13 (12.1%)	10 (16.1%)	1.101 (0.557-2.174)	0.782

a. Vasoactive agents include epinephrine, norepinephrine, isoproterenol, and dopamine during hospitalization. Bold values represent statistically significant associations (p < 0.05).

ICU, Intensive Care Unit; APACHE II, Acute Physiology and Chronic Health Evaluation II; CRAB, carbapenem-resistant *Acinetobacter baumannii*; CRKP, carbapenem-resistant *Klebsiella pneumoniae*; CRPA, carbapenem-resistant *Pseudomonas aeruginosa*; CR-GNB, Carbapenem-resistant Gram-negative Bacteria; MIC, minimum inhibitory concentration; CMS, Colistimethate Sodium; CBA, colistin base activity; HR, hazard ratio; CI, confidence interval; BL/BLI, β-lactam/β-lactamase inhibitor.

**Figure 3:**
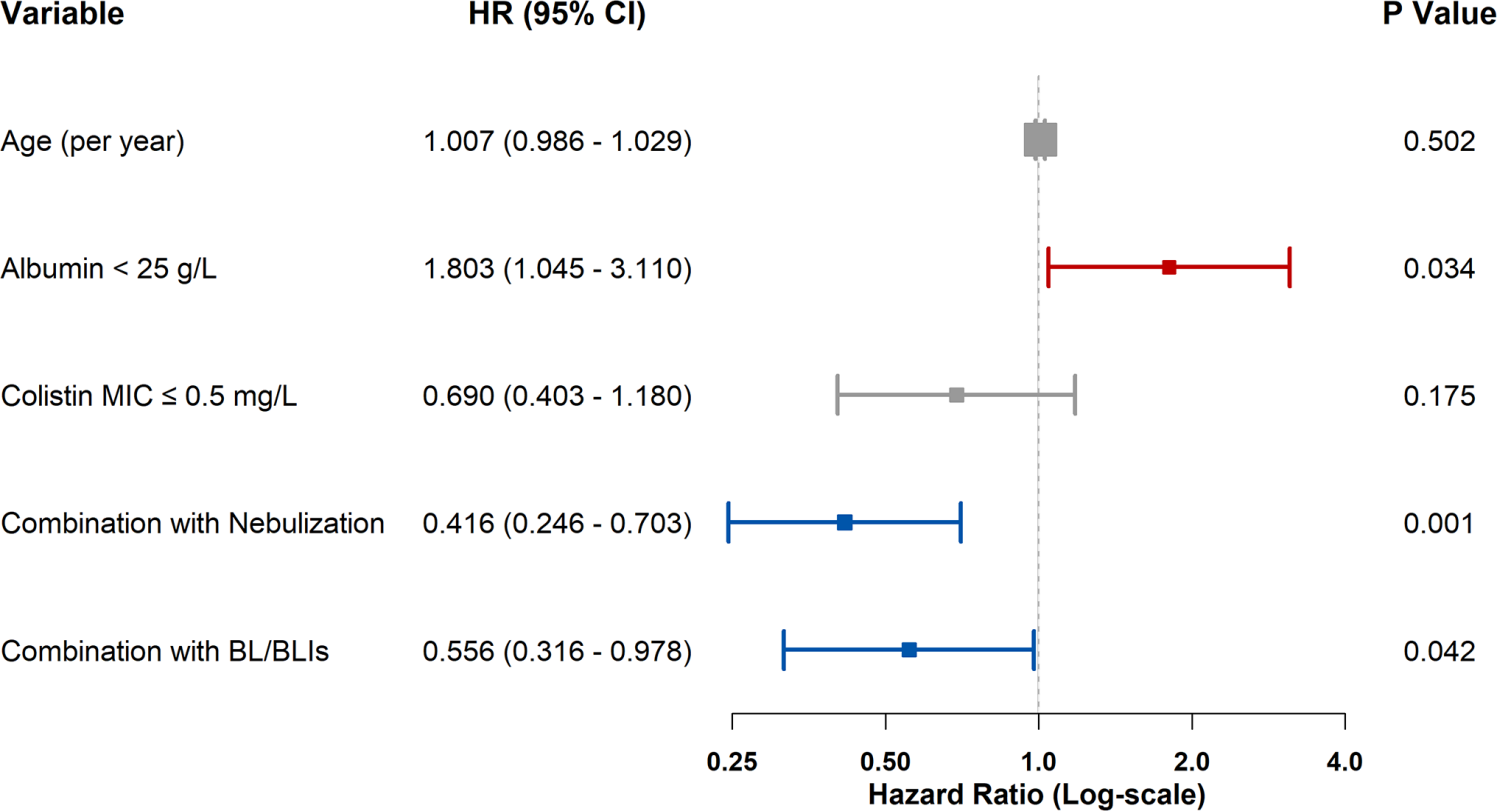
Forest plot of multivariable cox regression analysis for factors associated with all-cause mortality.

**Figure 4:**
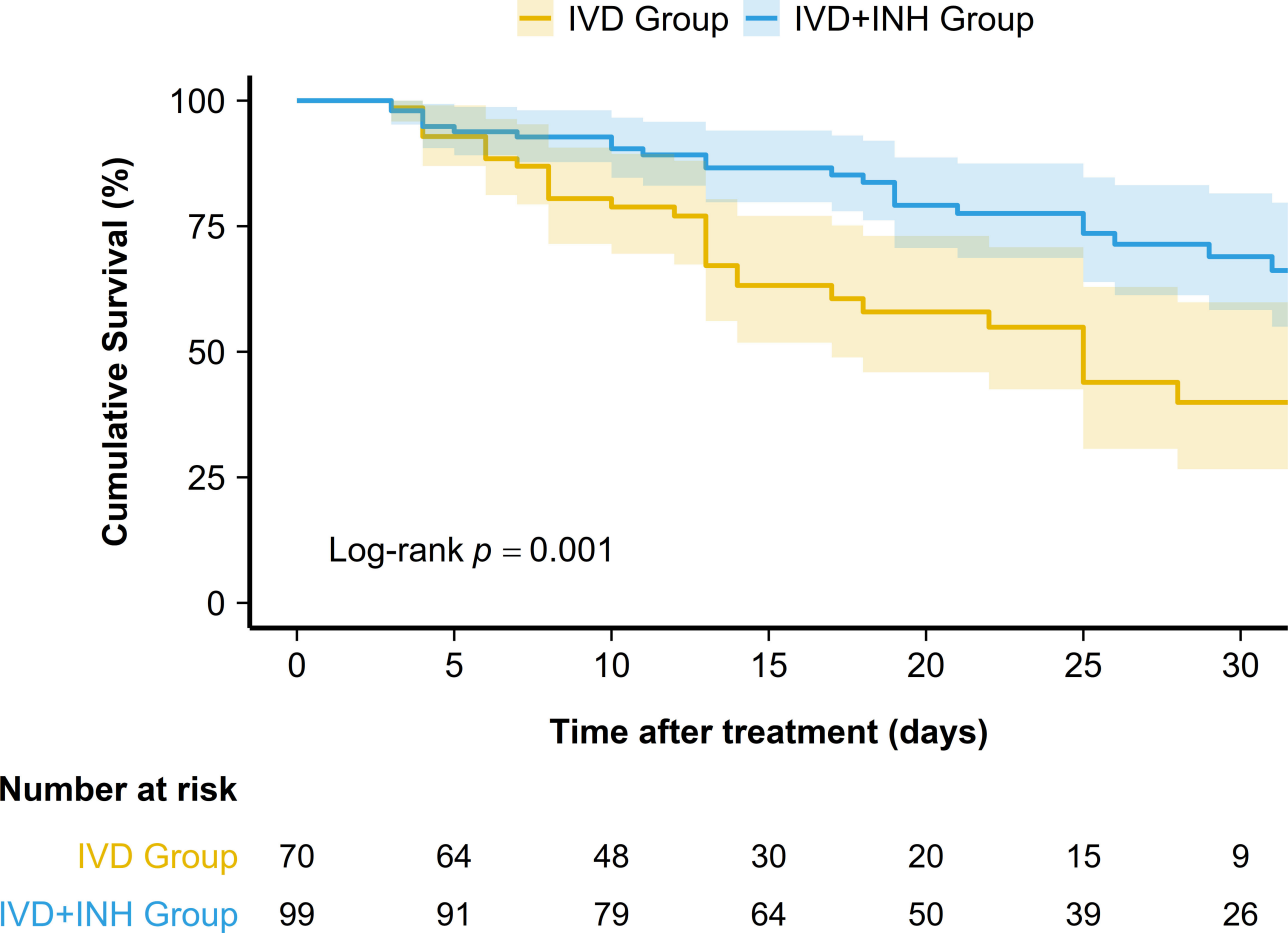
Kaplan-Meier curves for cumulative survival in patients treated with intravenous (IV) therapy alone (IVD group) versus IV plus nebulized therapy (IVD+INH group). IVD, intravenous drip; INH, inhaled.

As shown in **Figure 3**, multivariate Cox regression analysis indicated that adjunctive nebulization (adjusted hazard ratio [aHR] 0.416; 95% CI 0.246–0.703; *p* = 0.001) and combination with BL/BLIs (aHR 0.556; 95% CI 0.316–0.978; *p* = 0.042) were independently associated with a reduced risk of mortality. Meanwhile, serum albumin < 25 g/L remained an independent risk factor for mortality (aHR 1.803; 95% CI 1.045–3.110; *p* = 0.034). Kaplan-Meier survival analysis demonstrated that patients in the adjunctive nebulization group had a significantly higher cumulative survival rate compared to the non-nebulization group (Log-rank *p* = 0.001; **Figure 4**).

## 4 Discussion

CR-GNB infections significantly increase healthcare costs, prolong hospital stays, and raise in-hospital mortality, posing a critical global public health challenge that requires urgent intervention (Zhen et al., 2020; Dessemon et al., 2025). In this study, we present the clinical outcomes of CR-GNB infections treated with colistin-based regimens. The clinical efficacy, microbiological eradication, all-cause mortality, and AKI incidence were 40.8%, 50.3%, 36.7%, and 41.7%, respectively. Multivariate analysis showed that colistin MIC ≤ 0.5 mg/L and treatment duration were the primary factors correlated with clinical efficacy, while adjunctive nebulization and concomitant BL/BLIs were independently associated with a reduced risk of mortality.Current evidence regarding the clinical efficacy of CMS varies, with reported rates ranging from 54% to 83% (Katip et al., 2022; Lu and Mao, 2023). The relatively lower clinical efficacy (40.8%) observed in our study may be attributed to the conservative CMS maintenance dose. Although the median maintenance dose (2.83 mg CBA/kg/day) fell within the product label-recommended range, it was considerably lower than the optimal daily dose (300–360 mg CBA/day) recommended by the 2019 International Consensus Guidelines (Tsuji et al., 2019).

Under this conservative dosing strategy, treatment duration was a key factor linked to clinical success. Patients treated for 14–21 days achieved significantly better clinical outcomes than those receiving shorter courses (<14 days), with no additional benefit observed beyond 21 days (**Figure 2C**). This suggests that 14–21 days tended to be associated with more favorable outcomes, particularly when daily doses were restricted to the lower end of the range. This finding is consistent with previous studies. A study of 128 oncology patients with CRAB infections found that extended CMS treatment (≥ 14 days) was associated with more favorable clinical responses, higher microbiological eradication, and lower 30-day all-cause mortality compared with shorter treatment courses (Katip et al., 2021). Another study involving 374 critically ill patients with CRAB infections demonstrated that extended-duration polymyxin therapy significantly reduced 30-day mortality and improved both clinical and microbiological outcomes, supporting prolonged therapy in selected critically ill populations (Katip et al., 2023). Although prior research has established the importance of treatment duration, it did not further explore the impact of cumulative dose. Our analysis extends these findings by incorporating drug exposure quantification, showing that the median cumulative dose in the 14–21 days group (44.4 mg CBA/kg) fell within the 40–50 mg/kg range (**Figure 2B**). Cumulative dose-stratified analysis also identified the 40–50 mg/kg group as having the best clinical efficacy (**Figure 2D**). This consistency suggests that the clinical benefit of extended therapy may be associated with achieving this cumulative exposure window. Coupled with drug exposure, pathogen susceptibility constitutes another key determinant of efficacy. We identified colistin MIC ≤ 0.5 mg/L as an independent predictor of clinical response. As concentration−dependent antibiotics, polymyxins exert their bactericidal activity primarily through the *f*AUC_0-24h_/MIC ratio (Bergen et al., 2011; Zamri et al., 2025). When the *f*AUC_0-24h_ is constrained by conservative dosing, the probability of target attainment becomes highly sensitive to MIC, rendering a low MIC threshold essential for achieving the bactericidal target in this clinical setting.

In this study, we evaluated the dose–response relationship of CMS by stratifying patients according to daily maintenance dose, treatment duration, and cumulative exposure. Our findings identified a potential optimal cumulative dose window of 40–50 mg CBA/kg associated with improved clinical efficacy. From a PK/PD perspective, the bactericidal effect of polymyxins is largely determined by the fAUC0-24h/MIC ratio, which implies that conservative low-dose strategies restrict systemic drug exposure, making clinical outcomes highly sensitive to bacterial MIC. Although we provided a descriptive exposure–response analysis and PK/PD mechanism interpretation, further quantitative modeling with serial pharmacokinetic data remains necessary to establish more precise dosing recommendations for CMS in CR-GNB infections.

Multivariate analysis identified CMS monotherapy as an independent predictor of clinical failure, whereas combination therapy significantly improved microbiological eradication and patient survival. Polymyxins are known to exhibit heteroresistance, and monotherapy may be accompanied by an increased risk of resistance development (Mousavi et al., 2025). Consistent with this, a recent meta-analysis of 26 studies demonstrated that colistin-based combination therapy significantly enhances microbiological eradication compared with monotherapy (Yang et al., 2026). It is noteworthy that the observed association between combination therapy and favorable outcomes, particularly with BL/BLIs, might be partially explained by the high prevalence of CRAB (50.3%) in our cohort. In this context, sulbactam-based regimens possess unique intrinsic bactericidal activity against CRAB (Dorazio et al., 2025).

A key finding of our study was that adjunctive nebulization significantly reduced all-cause mortality. This survival benefit is likely consistent with the favorable pulmonary pharmacokinetics of inhaled therapy; unlike intravenous polymyxins that exhibit poor tissue penetration, nebulization bypasses the systemic circulation to deliver high drug concentrations directly to the infection site (Lee et al., 2024; Zhou et al., 2024; Gkoufa et al., 2022). Given the predominance of respiratory tract infections, this targeted delivery may represent a crucial intervention for optimizing outcomes in CR-GNB pneumonia.

This study has several limitations. First, the retrospective observational design was susceptible to confounding bias, as treatment choices may have been driven by disease severity and physician preference. Although multivariable analysis was used to adjust for major confounders, residual confounding could not be eliminated. Second, the analysis of treatment duration was prone to immortal time bias due to the fixed categorization of therapy length, which may have overestimated the benefit of prolonged treatment. The observed association between 14–21 days of treatment and favorable outcomes should be interpreted as correlational rather than causal. Third, the primary outcome included physician−assessed subjective improvement, which may introduce measurement bias despite standardized definitions. Fourth, the lack of therapeutic drug monitoring precluded individualized dose optimization. Fifth, the study cohort was dominated by respiratory tract infections, limiting the generalizability to other infection sites. Further large−scale prospective studies are warranted to optimize CMS therapy.

## 5 Conclusions

CMS demonstrated potential therapeutic value in the treatment of CR-GNB infections. Longer treatment durations and low colistin MIC were independent predictors of favorable clinical efficacy. Adjunctive nebulized CMS and combined BL/BLI regimens correlated with reduced all-cause mortality, particularly in patients with respiratory tract infections.

## Data Availability

The data analyzed in this study is subject to the following licenses/restrictions: The dataset contains sensitive patient information and is protected by privacy regulations. Access is restricted to authorized researchers only, in compliance with the Institutional Review Board (IRB) approval and patient confidentiality agreements. Requests to access these datasets should be directed to cljcarrie@163.com.
